# Genetic polymorphisms in the telomere length-related gene *ACYP2* are associated with the risk of colorectal cancer in a Chinese Han population

**DOI:** 10.18632/oncotarget.14219

**Published:** 2016-12-25

**Authors:** Fang Liu, Zhongguo Zhang, Yong Zhang, Yue Chen, Xiaoyu Yang, Jibin Li, Jiaxing Zhao

**Affiliations:** ^1^ Large-Scale Data Analysis Center of Cancer Precision Medicine, Cancer Hospital of China Medical University, Liaoning Cancer Hospital & Institute, Dadong District, Shenyang 110042, Liaoning Province, P R China; ^2^ Department of Colorectal Cancer Surgery, Cancer Hospital of China Medical University, Liaoning Cancer Hospital & Institute, Dadong District, Shenyang 110042, Liaoning Province, P R China; ^3^ Department of Pathology, Cancer Hospital of China Medical University, Liaoning Cancer Hospital & Institute, Dadong District, Shenyang 110042, Liaoning Province, P R China

**Keywords:** single nucleotide polymorphism (SNP), *ACYP2*, telomere length, colorectal cancer, association study

## Abstract

We investigated the association between single nucleotide polymorphisms (SNPs) in *ACYP2*, which has been associated with telomere length in several types of cancer, and the risk of CRC in a Chinese Han population. In a case-control study that included 247 cases and 300 healthy controls, 14 SNPs in *ACYP2* were selected and genotyped using the Sequenom MassARRAY platform. Odds ratios (ORs) and 95% confidence intervals (CIs) were calculated using unconditional logistic regression after adjusting for age and gender. We determined that rs843711 and rs843706 were associated with an increased risk of CRC (rs843711: OR = 1.376, 95% CI = 1.082-1.749, *p* = 0.009; rs843706: OR = 1.361, 95% CI = 1.069-1.733, *p* = 0.012). Additionally, rs6713088, rs843645, rs843711, and rs843706 were associated with an increased risk of CRC under additive and recessive models (*p* < 0.05). Finally, the “TTCTCGCC” and “CG” haplotypes decreased the risk of CRC, while the “AG” haplotype increase the risk of CRC. The association between rs843711 and CRC remained significant after Bonferroni correction for multiple comparisons (*p* ≤ 0.00036). Our data shed new light on the associations between genetic variants in the *ACYP2* gene and CRC susceptibility in a Chinese Han population.

## INTRODUCTION

Colorectal cancer (CRC) is the third most common cancer and the fourth leading cause of cancer deaths worldwide. The incidence of CRC is increasing each year [1]. Although the development of novel therapeutics has increased the 5-year survival rate to 90% for patients who are diagnosed at stage I, the prognosis of patients with advanced stage CRC is poor [2]. Therefore, the identification of epidemiological factors that influence the development of CRC will enable facilitate prevention and early detection, resulting in improve patient outcomes. Twin studies have shown that the contribution of inherited factors (mainly genetic) to the etiology of CRC is approximately 35% [3], which means that genetic factors significantly impact CRC susceptibility.

Telomeres are nucleoprotein complexes at the ends of eukaryotic chromosomes. These structures consist of noncoding TTAGGG repeats and associated telomere binding proteins. Telomeres maintain chromosome integrity and genomic stability by preventing nucleolytic degradation, chromosomal end-to-end fusion, and irregular recombination [4–6]. Telomere length is determined by a balance between processes that shorten and lengthen the telomere [7]. The average telomere length ranges from 10-15 kb in human somatic cells. Telomere length is reduced by 50-200 bp with each cell division [8]. During somatic cell replication, telomere length progressively shortens due to the inability of DNA polymerase to fully replicate the 3′ end of the DNA. In general, a critically short telomere length can trigger replicative senescence and cell death [9]. This can result in genomic instability and chromosomal abnormalities, which can promote carcinogenesis [10].

The *ACYP2* gene, located on chromosome 2p16.2, encodes a small cytosolic acylphosphatase enzyme that catalyzes the hydrolysis of carboxyl-phosphate bonds [11]. Genome wide association studies have demonstrated that genetic polymorphisms in *ACYP2* are associated with telomere length [12], which has led to studies of the association between *ACYP2* and various cancers. For example, rs11125529 in *ACYP2* was found to be associated with the risk of several hormone-related cancers (e.g. breast, ovarian, and prostate) in a European population [13, 14]. However, few studies have investigated the association between genetic variants in *ACYP2* and the risk of CRC. We performed a case-control study to analyze the association between 14 single nucleotide polymorphisms (SNPs) in *ACYP2* and the risk of CRC in a Chinese Han population.

## RESULTS

A total of 247 CRC cases (107 men and 140 women; mean age, 58.32 ± 12.75 years) and 300 controls (180 men and 120 women; mean age, 60.42 ± 5.14 years) were included in the study. The clinical characteristics of the cases and controls are shown in Table 1. There were no significant differences in the age and gender distributions between the case and control groups (*p* < 0.05). The minor allele frequencies (MAFs) of the analyzed SNPs in the case and control groups are shown in Table 2. All SNPs were in Hardy-Weinberg equilibrium (HWE) in the controls (*p* > 0.05) with the exception of rs843740, which was excluded from subsequent analyses. The MAFs of the SNPs in the control group were similar to those reported for the HapMap Asian population. Using chi-square tests, we determined that rs843711 was associated with a 1.376-fold increase in the risk of CRC (95% confidence interval [CI] = 1.082-1.749; *p* = 0.009). Similarly, rs843706 was associated with a significant increase in the risk of CRC (odds ratio [OR] = 1.361, 95% CI = 1.069-1.733; *p* = 0.012). No significant associations were detected between the other SNPs and CRC risk.

**Table 1:** Characteristics of the cases and controls included in the study

Variables	Case (N = 247)	Control (N = 300)	Total	*p* value
Sex, No. (%)				< 0.001^a^
Male	107 (43.3)	180 (60)	287 (52.5)	
Female	140 (56.7)	120 (40)	260 (47.5)	
Mean age ± SD	58.32 ± 12.75	60.42 ± 5.14		0.015^b^

^a^ The *p* value was calculated using Pearson's chi-square tests.

^b^ The *p* value was calculated using Welch's t tests.

SD, standard deviation.

**Table 2:** Allele frequencies in cases and controls and odds ratio estimates for colorectal cancer

SNP ID	Gene (s)	Band	Alleles A^a^/B	MAF		HWE *p*-value	ORs	95% CI	*p*-value
				Case	Control				
rs6713088	*ACYP2*	2p16.2	G/C	0.429	0.377	0.623	1.243	0.974-1.585	0.080
rs12621038	*ACYP2*	2p16.2	T/C	0.411	0.450	0.415	0.852	0.669-1.084	0.193
rs1682111	*ACYP2*	2p16.2	A/T	0.309	0.333	0.604	0.894	0.692-1.155	0.391
rs843752	*ACYP2*	2p16.2	G/T	0.287	0.248	0.877	1.216	0.929-1.592	0.154
rs10439478	*ACYP2*	2p16.2	C/A	0.409	0.425	0.237	0.937	0.736-1.193	0.597
rs843645	*ACYP2*	2p16.2	G/T	0.285	0.237	0.749	1.283	0.978-1.683	0.072
rs11125529	*ACYP2*	2p16.2	A/C	0.198	0.170	0.411	1.208	0.889-1.643	0.227
rs12615793	*ACYP2*	2p16.2	A/G	0.209	0.185	0.254	1.161	0.860-1.565	0.329
rs843711	*ACYP2*	2p16.2	T/C	0.494	0.415	0.287	1.376	1.082-1.749	0.009*
rs11896604	*ACYP2*	2p16.2	G/C	0.213	0.177	1.000	1.258	0.931-1.699	0.134
rs843706	*ACYP2*	2p16.2	A/C	0.496	0.419	0.341	1.361	1.069-1.733	0.012*
rs17045754	*ACYP2*	2p16.2	C/G	0.192	0.172	0.547	1.149	0.844-1.564	0.378
rs843740	*ACYP2*	2p16.2	A/G	0.453	0.403	< 0.001^#^	1.227	0.965-1.561	0.095
rs843720	*ACYP2*	2p16.2	G/T	0.358	0.358	0.451	0.999	0.780-1.282	0.999

MAF, minor allelic frequency; HWE, Hardy-Weinberg Equilibrium; OR, odds ratio; CI: confidence interval.

^a^ Minor allele; ^#^ HWE *p*-value ≤ 0.05 was excluded; *p value ≤ 0.05.

Bonferroni correction was performed with *p* ≤ 0.00036 (0.05/14) considered significant.

The genotype frequencies of the *ACYP2* polymorphisms are shown in Table 3. Compared to the CC genotype, the frequency of the GG genotype of rs6713088 polymorphism in the case group significantly differed from the controls (GG vs. CC: OR = 1.750, 95% CI = 1.032-2.967; *p* = 0.038), suggesting that rs6713088 increased the risk of CRC. Similarly, compared to individuals with the CC genotype of rs843711, individuals with the TT genotype had a significantly increased risk of CRC (TT vs. CC: OR = 2.007, 95% CI = 1.218-3.308; *p* = 0.006). Individuals with the AA genotype of rs843706 also had an increased risk of CRC compared to those with the CC genotype (AA vs. CC: OR = 1.971, 95% CI = 1.184-3.280; *p* = 0.009).

**Table 3:** Genotype distributions of the SNPs and their associations with the risk of colorectal cancer

SNP ID	Alleles A^a^/B	Genotype	No. (frequency)		Without adjustment		With adjustment	
			Case (%)	Control (%)	OR (95% CI)	*P*	OR (95% CI)	*P*^b^
rs6713088	G/C	CC	80 (32.5)	114 (38)	1		1	
		GG	45 (18.3)	40 (13.3)	1.603(0.960-2.678)	0.071	1.750 (1.032-2.967)	0.038*
		GC	121 (49.2)	146 (48.7)	1.181 (0.813-1.716)	0.383	1.232 (0.840-1.807)	0.286
rs12621038	T/C	CC	86 (34.9)	94 (31.4)	1		1	
		TT	42 (17.1)	64 (21.4)	0.717 (0.441-1.167)	0.181	0.686 (0.417-1.129)	0.138
		TC	118 (48)	141 (47.2)	0.915 (0.625-1.339)	0.647	0.859 (0.581-1.271)	0.447
rs1682111	A/T	TT	117 (47.6)	131 (43.7)	1		1	
		AA	23 (9.3)	31 (10.3)	0.831 (0.459-1.505)	0.541	0.877 (0.478-1.608)	0.671
		AT	106 (43.1)	138 (46)	0.860 (0.603-1.227)	0.406	0.894 (0.621-1.286)	0.545
rs843752	G/T	TT	124 (50.4)	170 (56.7)	1		1	
		GG	19 (7.7)	19 (6.3)	1.371 (0.697-2.697)	0.301	1.385 (0.692-2.77)	0.358
		GT	103 (41.9)	111 (37)	1.272 (0.893-1.813)	0.183	1.322 (0.920-1.901)	0.132
rs10439478	C/A	AA	83 (33.6)	104 (34.8)	1		1	
		CC	38 (15.4)	59 (19.7)	0.807 (0.490-1.330)	0.401	0.712 (0.427-1.190)	0.195
		CA	126 (51)	136 (45.5)	1.161 (0.797-1.692)	0.438	1.118 (0.760-1.645)	0.570
rs843645	G/T	TT	126 (51.2)	176 (58.7)	1		1	
		GG	20 (8.1)	18 (6)	1.552 (0.789-3.053)	0.203	1.622 (0.811-3.246)	0.172
		GT	100 (40.7)	106 (35.3)	1.318 (0.923-1.882)	0.129	1.380 (0.958-1.988)	0.084
rs11125529	A/C	CC	160 (64.8)	204 (68)	1		1	
		AA	11 (4.5)	6 (2)	2.338 (0.846-6.457)	0.101	2.631 (0.933-7.417)	0.067
		AC	76 (30.7)	90 (30)	1.077 (0.745-1.557)	0.695	1.003 (0.686-1.465)	0.988
rs12615793	A/G	GG	156 (63.1)	196 (65.3)	1		1	
		AA	12 (4.9)	7 (2.4)	2.154 (0.828-5.600)	0.116	2.384 (0.899-6.314)	0.081
		AG	79 (32)	97 (32.3)	1.023 (0.711-1.472)	0.901	0.953 (0.655-1.385)	0.799
rs843711	T/C	CC	67 (27.2)	98 (32.7)	1		1	
		TT	64 (26)	47 (15.7)	1.992 (1.222-3.245)	0.006*	2.007 (1.218-3.308)	0.006*
		TC	115 (46.8)	155 (51.6)	1.085 (0.732-1.608)	0.684	1.073 (0.718-1.603)	0.732
rs11896604	G/C	CC	156 (63.2)	203 (67.7)	1		1	
		GG	14 (5.7)	9 (3)	2.024 (0.854-4.798)	0.109	2.219 (0.915-5.384)	0.078
		GC	77 (31.1)	88 (29.3)	1.139 (0.786-1.649)	0.492	1.050 (0.718-1.537)	0.800
rs843706	A/C	CC	61 (25.3)	96 (32.2)	1		1	
		AA	59 (24.5)	48 (16.1)	1.934 (1.175-3.183)	0.009*	1.971 (1.184-3.280)	0.009*
		AC	121 (50.2)	154 (51.7)	1.237 (0.829-1.844)	0.298	1.256 (0.835-1.888)	0.274
rs17045754	C/G	GG	164 (66.4)	204 (68)	1		1	
		CC	12 (4.8)	7 (2.3)	2.132 (0.821-5.539)	0.120	2.295 (0.867-6.077)	0.095
		CG	71 (28.8)	89 (29.7)	0.992 (0.683-1.442)	0.968	0.914 (0.622-1.342)	0.646
rs843720	G/T	TT	100 (40.5)	120 (40)	1		1	
		GG	30 (12.1)	35 (11.7)	1.029 (0.590-1.792)	0.921	1.009 (0.573-1.780)	0.974
		GT	117 (47.4)	145 (48.3)	0.968 (0.675-1.388)	0.861	1.021 (0.706-1.476)	0.914

^a^ Minor alleles, OR: Odds ratio, CI: Confidence interval;

^b^: *p* values were calculated using unconditional logistic regression after adjusting for sex and age;

*: *p* ≤ 0.05.

Bonferroni correction was performed with *p* ≤ 0.00036 (0.05/14) considered significant.

We assumed that the minor allele of each SNP was a risk factor compared to the wild-type allele. Three genetic models (dominant, recessive, and additive) were applied to analyze the associations between the SNPs and CRC risk using an unconditional logistic regression analysis with adjustments for age and gender (Table 4). We found that the minor allele (G) of rs6713088 and rs843645 was associated with an increased risk of CRC under the additive model (rs6713088: OR = 1.304, 95% CI = 1.012-1.681; *p* = 0.04. rs843645: OR = 1.322, 95% CI = 1.001-1.746; *p* = 0.049). The minor allele (T) of rs843711 was associated with an increased risk of CRC under the additive model (OR = 1.38, 95% CI = 1.077-1.768; *p* = 0.011) and recessive model (OR = 1.921, 95% CI = 1.247-2.960; *p* = 0.003). Additionally, the minor allele (A) of rs843706 was associated with a significantly increased risk of CRC under the additive model (OR = 1.39, 95% CI = 1.079-1.791; *p* = 0.011) and recessive model (OR = 1.703, 95% CI = 1.101-2.633; *p* = 0.017). However, only rs843711 remained significant after Bonferroni correction for multiple comparisons (*p* ≤ 0.00036).

**Table 4:** Logistic regression analysis of the association between the SNPs and colorectal cancer (adjusted for sex and age)

SNP ID	Minor allele	Additive model			Dominant model			Recessive model		
		OR	95% CI	*p*-value	OR	95% CI	*p*-value	OR	95% CI	*p*-value
rs6713088	G	1.304	1.012-1.681	0.040*	1.340	0.932-1.928	0.115	1.549	0.962-2.496	0.072
rs12621038	T	0.832	0.652-1.062	0.140	0.805	0.558-1.162	0.246	0.750	0.482-1.167	0.202
rs1682111	A	0.920	0.706-1.199	0.538	0.891	0.630-1.260	0.513	0.927	0.520-1.654	0.798
rs843752	G	1.243	0.942-1.640	0.124	1.331	0.941-1.884	0.106	1.230	0.626-2.417	0.548
rs10439478	C	0.885	0.692-1.133	0.333	0.992	0.690-1.428	0.967	0.667	0.421-1.057	0.085
rs843645	G	1.322	1.001-1.746	0.049*	1.415	0.999-2.004	0.051	1.421	0.723-2.795	0.308
rs11125529	A	1.183	0.862-1.624	0.299	1.097	0.761-1.581	0.618	2.628	0.938-7.364	0.066
rs12615793	A	1.136	0.833-1.549	0.420	1.043	0.727-1.496	0.819	2.422	0.921-6.365	0.073
rs843711	T	1.380	1.077-1.768	0.011*	1.288	0.882-1.879	0.190	1.921	1.247-2.960	0.003*
rs11896604	G	1.216	0.898-1.649	0.207	1.151	0.801-1.655	0.447	2.187	0.908-5.265	0.081
rs843706	A	1.390	1.079-1.791	0.011*	1.425	0.968-2.097	0.073	1.703	1.101-2.633	0.017*
rs17045754	C	1.108	0.809-1.516	0.524	1.009	0.699-1.458	0.960	2.356	0.896-6.197	0.082
rs843720	G	1.010	0.779-1.308	0.943	1.018	0.717-1.447	0.919	0.998	0.587-1.698	0.995

OR, odds ratio; CI: confidence interval.

*p ≤ 0.05.

Bonferroni correction was performed with *p* ≤ 0.00036 (0.05/14) considered significant.

**Figure 1:**
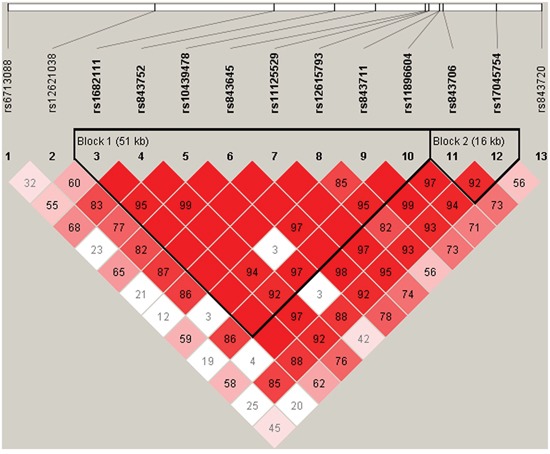
D’ linkage map for the 13 SNPs in ACYP2 A standard color scheme was used to display linkage disequilibrium (LD), with bright red corresponding to strong LD (LOD = 2, *D*’ = 1), white corresponding to no LD (LOD < 2, *D*’ < 1), and pink/red (LOD = 2, *D*’ < 1) and blue (LOD < 2, *D*’ = 1) corresponding to intermediate LD.

We further characterized the SNPs in *ACYP2* SNPs using linkage disequilibrium (LD) and haplotype analyses. Pairwise LD was calculated between all 13 SNPs and the haplotype structure of the *ACYP2* gene was analyzed (D’ and r^2^). Haplotype blocks were divided using the D’ confidence interval method. Adjacent SNPs with D’ values and 95% CIs between 0.70-0.98 were classified as the same haplotype block. Two LD blocks were detected in the control group (Figure 1). Block 1 consisted of 8 closely linked SNPs (rs1682111, rs843752, rs10439478, rs843645, rs11125529, rs12615793, rs843711, and rs11896604) and block 2 consisted of two linked SNPs (rs843706 and rs17045754).

Finally, a haplotype-based association study was performed to evaluate the relationship between *ACYP2* haplotypes and risk of CRC (Table 5). The “TTCTCGCC” haplotype was associated with a decreased risk of CRC (OR = 0.719; 95% CI, 0.529-0.978, *p* = 0.035). This haplotype consisted of rs1682111, rs843752, rs10439478, rs843645, rs11125529, rs12615793, rs843711, and rs11896604. Additionally, the “AG” haplotype of rs843706 and rs17045754 was associated with an increased risk of CRC (OR = 1.377; 95% CI, 1.044-1.815, *p* = 0.023). In contrast, the “CG” haplotype was associated with a decreased risk of CRC (OR = 0.732; 95% CI, 0.568-0.944, *p* = 0.016).

**Table 5:** *ACYP2* haplotype frequencies and the association with the risk of colorectal cancer in cases and controls

Block ID	SNPs	Haplotype	Freq (case)	Freq (control)	χ2	Pearson's *p*	OR	95% CI		*p*-adj
1	rs1682111, rs843752, rs10439478, rs843645, rs11125529, rs12615793, rs843711, rs11896604	TGAGCGTC	0.279	0.231	3.279	0.070	1.325	1.001	1.754	0.050
		ATATCGCC	0.303	0.327	0.674	0.412	0.930	0.713	1.213	0.592
		TTCTAATG	0.201	0.172	1.506	0.220	1.184	0.862	1.626	0.298
		TTCTCGCC	0.182	0.227	3.289	0.070	0.719	0.529	0.978	0.035*
		TTCTCACC	0.008	0.015	1.092	0.297	0.542	0.160	1.837	0.326
2	rs843706, rs17045754	AC	0.185	0.166	0.636	0.425	1.107	0.805	1.523	0.532
		AG	0.311	0.253	4.431	0.035	1.377	1.044	1.815	0.023*
		CG	0.504	0.576	5.469	0.019	0.732	0.568	0.944	0.016*
		TA	0.171	0.139	2.144	0.143	1.294	0.925	1.811	0.133

OR: odd ratio; CI: confidence interval.

*p*-adj: *p*-values were adjusted for sex and age;

*p value ≤ 0.05.

Bonferroni correction was performed, with *p* ≤ 0.00036 (0.05/14).

## DISCUSSION

We investigated the associations between 14 SNPs in the *ACYP2* gene and the risk of CRC in a Chinese Han population. We determined that four SNPs (rs6713088, rs843645, rs843711, and rs843706) were associated with an increased risk of CRC. Additionally, we demonstrated that three haplotypes in *ACYP2* were associated with CRC risk.

Previous studies have revealed an association between the *ACYP2* gene and cancer cell metabolism (i.e. pyruvate metabolism and glycolysis/gluconeogenesis) [15, 16]. Cancer cell survival and proliferation is associated with glucose uptake and altered expression of several metabolic intermediates in the glycolysis/gluconeogenesis pathway such as phosphofructokinase (PFK) and pyruvate kinase [17, 18]. PFK is essential for glycolysis and is a possible target for anticancer drugs. The transition of cancer cells to a highly proliferative state is correlated with reduced PFK expression [19]. Furthermore, pyruvate metabolism can generate lactate or alanine and is accompanied by the conversion of NADH to NAD^+^, which could provide substrates and energy for cancer cells to drive proliferation and differentiation [20]. Thus, stimulation of the pyruvate metabolic pathway may promote malignant transformation [18, 21]. We found that SNPs in *ACPY2* were associated with CRC risk. Our data suggest that *ACPY2* may play an important role in regulating glucose and pyruvate metabolism in CRC patients. However, the mechanistic details have not yet been elucidated.

Telomeres have a critical role in maintaining genomic stability [22, 23]. Telomere lengths become shorter following each cell division. Therefore, telomere length could serve as a marker of cellular and biological age [24]. It is possible that shortened telomeres may predispose individuals to certain diseases. Genome-wide association studies have demonstrated that rs11125529 in the *ACYP2* gene was associated with reduced telomere length [12, 25]. Although we did not observe an association between rs11125529 and CRC risk in our study population, we did find that rs6713088, rs843645, rs843711, and rs843706 were associated with CRC risk. Furthermore, we found that the “TTCTCGCC” and “CG” haplotypes were protective against CRC while the “AG” haplotype may increase the risk of CRC. These results suggested that genetic variations in the *ACYP2* gene may impact CRC risk by influencing telomere length.

The Bonferroni correction is commonly used to address false discovery rates resulting from multiple comparisons. We found that only the association between rs843711 and CRC was significant after Bonferroni correction, while rs843706, rs6713088, and rs843645 did not have a significant association with CRC. This may be due to our strict SNP filtering criteria and small sample size. The Bonferroni correction adjusts the value of alpha based on the number of tests performed and is relatively conservative. In some cases, truly significant differences may be deemed non-significant as a result of type II errors [26].

Our study had several intrinsic limitations. For example, CRC is a heterogeneous disease, and alcohol and tobacco consumption are important risk factors for CRC. Because our study had a relatively small size, and it did not incorporate data regarding alcohol and tobacco consumption, we could not explore the interactions between genetic polymorphisms and environmental factors in CRC patients. Therefore, the relationship between *ACYP2* polymorphisms and drinking and smoking status in CRC must be evaluated in future studies.

In summary, our results indicate that SNPs in the *ACYP2* gene are associated with CRC in a Chinese Han population. These SNPs may serve as a prognostic biomarkers for CRC in the Chinese population. Future studies will focus on elucidating the function of *ACYP2* in CRC, which could be important for CRC prevention or early detection, and for improving patient prognosis.

## MATERIALS AND METHODS

### Study participants

All participants in our study were Han Chinese. A total of 247 patients and 300 controls were consecutively recruited between January 2011 and December 2014 at the LiaoNing Cancer Hospital and Institute, Affiliated Tumor Hospital of China Medical University, China. There were no gender, age, or disease stage restrictions for case recruitment. All cases were previously healthy individuals. The diagnosis of CRC was confirmed by histopathological examination. The control subjects had personal history of malignancy or chronic disease. We excluded patients who underwent radiotherapy or chemotherapy, as well as controls with chronic diseases. All participants in the current study were not related by blood.

### Clinical data and demographic information

We used a standard epidemiological questionnaire and in-person interview to collect personal data including age, gender, residential region, education status, and family history of cancer. The case information was collected through consultation with the treating physicians or from medical chart reviews. All of the participants provided written informed consent. The Human Research Committee for Approval of Research Involving Human Subjects, LiaoNing Cancer Hospital and Institute, Affiliated Tumor Hospital of China Medical University, approved the use of human blood samples in this study.

### SNP selection and genotyping

Among the 14 SNPs we selected, rs11125529 was chosen based on previously published polymorphisms associated with telomere length [12]. The others were randomly selected. All of the SNPs had MAFs > 5% in the HapMap Chinese Han Beijing population. DNA was extracted from whole blood samples using the GoldMag-Mini Whole Blood Genomic DNA Purification Kit (GoldMag Co. Ltd. Xi’an City, China). Quantification of the extracted DNA was performed using a NanoDrop 2000 (Thermo Scientific, Waltham, Massachusetts, USA). The multiplexed SNP MassEXTENDED assay was designed using the Sequenom MassARRAY Assay Design 3.0 Software [27]. Genotyping was performed on a Sequenom MassARRAY RS1000 platform using the manufacturer's protocol. The PCR primers for each SNP are shown in Table 6. Data management and analysis was performed using the Sequenom Typer 4.0 Software [27, 28].

**Table 6:** Primers used in this study

SNP_ID	1st_PCRP	2st_PCRP	UEP_SEQ
rs6713088	ACGTTGGATGACACACACAGACTCCTTCAC	ACGTTGGATGGTCACCAAAACACGTAATG	gaggcCAGAATGGTCCACTAGAGA
rs12621038	ACGTTGGATGATTGTGCTAGGCACTTTAGG	ACGTTGGATGGGCATAAGTTTTATTGCCTC	ccATTGCCTCAGCTAGACT
rs1682111	ACGTTGGATGGAATTGCTGGGTTATTTGGC	ACGTTGGATGGCCAGTGGGAATGCAAAATG	tgtcATGCAAAATGAAACAGACACTT
rs843752	ACGTTGGATGTCCTCTTTTCAGAAACCTGC	ACGTTGGATGGAGACAACATAATGGAGGTC	cGAGTTTGGGTTTGAGGT
rs10439478	ACGTTGGATGTAGCACAAGACCTACACTGG	ACGTTGGATGCTACACTCTCCAGAGGAATG	TTGCTGTTTTCCCAGAA
rs843645	ACGTTGGATGACAGTGCCTTTAGCAAGGTG	ACGTTGGATGGAAATCTGAATACCACCTAC	TCATAGGCACTACTGTATC
rs11125529	ACGTTGGATGCCGAAGAAAAGAAGATGAC	ACGTTGGATGGAGCTTAGTTGTTTACAGATG	AGAAAAGAAGATGACTAAAACAT
rs12615793	ACGTTGGATGATCTTGGCCCTTGAAGAA	ACGTTGGATGTTTGAGCTTAGTTGTTTAC	AAATTGAGTGACAAATATAAACTAC
rs843711	ACGTTGGATGTGCCTTGTGGGAATTAGAGC	ACGTTGGATGGACAAAGGACCTTACAACTC	gggaTCAGGGAACCAGTGCAAA
rs11896604	ACGTTGGATGTGTCTCTGACCTAGCATGTA	ACGTTGGATGAAGTCAGAATAGTGCTTAC	GTTAAGCTTGCAAGGAG
rs843706	ACGTTGGATGTGAATAACTTGGTCTTATC	ACGTTGGATGTGAAAGCCATAAATATTTTG	cACTTGGTCTTATCTGATGC
rs17045754	ACGTTGGATGCTGTAAAAGTTCTGGCATGG	ACGTTGGATGGAAATCAGGGATATTAGTGC	caggTATTCAGCTTCCTAGAGTTA
rs843740	ACGTTGGATGTCACAGACCCCCATAGTTAG	ACGTTGGATGTGAGGAAACTGAAGTTTAGC	ccctcTTGCTTCTTGGGGCCTAACA
rs843720	ACGTTGGATGCTTCACAACACTCCTGTAAG	ACGTTGGATGAGTCAGAGCTAGACCTCTGG	ccccAATCTGTCTCAGGGTCTT

### Statistical analysis

We used Microsoft Excel and the SPSS 18.0 statistical package (SPSS, Chicago, IL, USA) to perform statistical analyses. All *p* values presented in this study were two sided, and *p* = 0.05 was considered the cutoff for statistical significance. Differences in the characteristics of the case and control study populations were analyzed using chi-square tests for categorical variables and Welch's t tests for continuous variables. In all analyses, the lower frequency allele was considered to be the ‘risk’ allele. Control genotype frequencies for each SNP were tested for departure from HWE using Fisher's exact tests. Allele and genotype frequencies in the cases and controls were compared using chi-square tests [29]. Three models (additive, dominant, and recessive) were used to assess the association between each genotype and the risk of CRC. The effects of the polymorphisms on the risk of CRC were expressed as ORs with 95% CIs, which were calculated using unconditional logistic regression analysis after adjusting for age and gender [30]. Finally, LD patterns and haplotypes were evaluated using the Haploview software package (version 4.2) [31]. Bonferroni correction was performed on all *p* values, and the threshold for statistical significance was set at *p* ≤ 0.00036 (0.05/14).
